# Multiple myeloma in adolescents and young adults(AYA): global epidemiological trends, risk factors, and future projections (1990–2040)

**DOI:** 10.1007/s10552-026-02142-3

**Published:** 2026-02-19

**Authors:** Rupayan Kundu, Ankush Mukhopadhyay, Nivedita Sarkar, Niladri Kal, Tuhin Subhra Pal, Kriti Soni, Sudipto Mukherjee

**Affiliations:** 1 Department of Internal Medicine, Cleveland Clinic Foundation, 9500 Euclid Ave, Cleveland, OH USA; 2 Nil Ratan Sircar Medical College, Kolkata, India; 3 Dr. RML Hospital, New Delhi, India; 4 Department of Statistics, Texas A&M University, College Station, TX USA; 5 Department of Biological Sciences, Indian Institute of Science Education and Research, Kolkata, West Bengal India; 6 Department of Internal Medicine, Sardar Patel Medical College, Bikaner, India; 7 Department of Hematology and Medical Oncology, Cleveland Clinic Taussig Cancer Institute, Cleveland, OH USA

**Keywords:** Adolescents and Young Adults, AYA, Multiple Myeloma, MM, Cancers in adolescents and young adults, Epidemiology, Cancer, Early onset cancer, Incidence, Death, 1990, 2021, 2040, Projection

## Abstract

**Introduction:**

Multiple myeloma (MM) is a hematological malignancy with increasing incidence among adolescents and young adults (AYAs) aged 15 to 39 years. This trend presents unique challenges related to long-term treatment effects, fertility, and late-onset complications. Risk factors include obesity, diabetes, environmental exposures, and genetic predispositions, with a higher incidence observed in males and individuals of African-American descent. This study aims to analyze global trends in MM incidence and mortality among AYAs from 1990 to 2021 and project future trends through 2040.

**Methods:**

Using data from the Global Burden of Disease (GBD) 2021 study, we analyzed the age-specificincidence rate (IR), and death rate (DR) per 100,000 patient-years of AYA MM. Statistical modeling, including two sample t-test were done to estimate the standard deviations among each group, which is plugged into the denominator to compute the statistic. Auto-Regressive Integrated Moving Average (ARIMA) and Exponential Smoothing State Space (ETS) models were applied for projections.

**Results:**

In 2021, the global AYA MM incidence was 2536 cases (IR 0.09/100,000 patient-years), and deaths totaled 1579 (DR 0.05/100,000 patient-years). From 1990 to 2021, IR and DR increased (annual percentage change (APC) of 0.84 for IR and 0.60 for DR). Males exhibited higher rates and faster increases. High-middle SDI regions had the highest IR and DR, with Mauritius recording the highest national rates. High BMI contributed to 6.52% of MM-related deaths in 2021. Projections indicate rising IR and DR globally through 2040.

**Conclusion:**

The increasing burden of AYA MM necessitates early detection, targeted treatments, and preventive measures addressing obesity and other modifiable risk factors. Reducing regional disparities through improved healthcare access is essential to mitigate the growing impact of MM.

**Supplementary Information:**

The online version contains supplementary material available at 10.1007/s10552-026-02142-3.

## Introduction

Multiple myeloma (MM) is a hematological malignancy characterized by the proliferation of malignant plasma cells in the bone marrow. Although traditionally viewed as a disease predominantly affecting older adults, recent epidemiological studies have highlighted a concerning trend of increasing incidence among adolescents and young adults (AYAs) aged 15 to 39 years.[1] However, AYAs face unique challenges, including the risk of late-onset adverse events and the impact of treatment on fertility and long-term health. This demographic shift raises critical questions about the underlying risk factors and the biological mechanisms that contribute to the development of MM in younger populations [2, 3].

The incidence of MM has been observed to vary significantly based on demographic factors such as age, sex, and ethnicity. Notably, males and individuals of African-American descent exhibit higher incidence rates compared to their female and Caucasian counterparts [4, 5] The increasing recognition of MM in AYAs necessitates an exploration of the unique risk factors that may contribute to this trend. Factors such as obesity, diabetes, and environmental exposures have been implicated in the pathogenesis of MM, with obesity being a well-documented risk factor that correlates with increased incidence [6–8]. Furthermore, lifestyle factors, including tobacco and alcohol use, have also been associated with elevated risk, although the evidence remains inconsistent [9].

The epidemiological landscape of MM is further complicated by the interplay of genetic predispositions and environmental influences. Studies have suggested that certain genetic variants may heighten susceptibility to MM, particularly in the context of other comorbid conditions such as type 2 diabetes mellitus [10] Additionally, environmental factors, including occupational exposures to specific chemicals, have been linked to increased risk, although the findings are often heterogeneous across different populations [11] Understanding these multifaceted risk factors is crucial for developing targeted prevention strategies and improving outcomes for young patients diagnosed with MM; however, among these, obesity represents a key modifiable risk factor with measurable population-level impact and is therefore a central focus of the present analysis.

In light of these trends, this paper aims to provide a comprehensive overview of the epidemiological trends in the incidence, mortality, and associated risk factors of AYA MM from 1990 to 2021, with projections extending to 2040. By synthesizing existing literature and analyzing demographic data, this study seeks to illuminate the evolving landscape of MM in younger populations and identify critical areas for future research and intervention.

## Methods

### Global burden of disease study

The GBD study offers comprehensive global, regional, and national estimates on the burden of diseases, injuries, and risk factors by consolidating all available data. GBD 2021 provides estimates for mortality, incidence, and disability-adjusted life years across 371 diseases and injuries, 288 causes of death, and 88 risk factors in 204 countries and territories. The methods for data collection in this research have been thoroughly detailed in prior publications. Rates per 100,000 population were standardized for age based on the GBD world population. Estimates were presented with 95% uncertainty intervals (95% UIs) to account for potential biases, measurement errors, and modeling uncertainties. This study complies with the Guidelines for Accurate and Transparent Health Estimates Reporting (GATHER) statement.[12].

### Case definition and data source

Adolescent and young adult (AYA) multiple myeloma (MM) is defined as MM in the age group of 15–39 years. MM is categorized according to the International Classification of Diseases **(**ICD) 9 and 10 codes: C88-C90.32 in ICD 10 and 203-203.9 in ICD 9. Data on AYA MM were retrieved from the Global Burden of Disease Study 2021. Incidence and deaths, along with their rates per 100,000 patient-years, were used to measure the burden of AYA MM. Global, regional, and national analyses were performed for the period between 1990 and 2021. Further sub-analyses were conducted based on age group, sex, and sociodemographic index (SDI).

### SDI regions

The SDI, ranging from 0 to 1, serves as a composite indicator of development and social well-being, incorporating total fertility rate, per capita income, and average educational attainment. Regions were stratified by Sociodemographic Index (SDI), which ranged from 0 (minimal development) to 1 (maximal development) and was classified as high (> 0.715), high-middle (0.625 to 0.0715), middle (0.558 to 0.624), low-middle (0.378 to 0.0557), and low (< 0.378) SDI regions.

### 21 GBD regions

The world has been divided into 21 GBD regions. The regions are: Andean Latin America, Australasia, Caribbean, Central Asia, Central Europe, Central Latin America, Central Sub-Saharan Africa, East Asia, Eastern Europe, Eastern Sub-Saharan Africa, High-income Asia Pacific, High-income North America, North Africa and Middle East, Oceania, South Asia, Southeast Asia, Southern Latin America, Southern Sub-Saharan Africa, Tropical Latin America, Western Europe, and Western Sub-Saharan Africa.

### Risk factors

This database identifies high body-mass index (BMI) as a risk factor associated with deaths of AYA MM. Detailed definitions of these risk factors and the estimation of the proportions of the AYA MM burden attributable to them have been previously described. The GBD 2021 utilized the widely accepted comparative risk assessment methodology to calculate the attributable burden for each risk factor.

Statistical analysis and estimation of disease burden of AYA MM.

We obtained data from the GBD 2021 database (https://vizhub.healthdata.org/gbd-results/). In our current research, we utilized annual incidence and mortality, along with their corresponding age-specific rates (AsR) per 100,000 individuals, to represent the burden of adolescents and young adults (AYAs) with multiple myeloma (MM). The methodological framework and protocols for Global Burden of Disease (GBD) research have been comprehensively described in earlier studies. [13, 14].

The DisMod MR-2.1 model, a Bayesian meta-regression approach, was employed to estimate the non-fatal burden of AYA MM. This process involved setting specific priors: (i) a minimum remission rate of 3 and a maximum of 5, corresponding to an average disease duration of 3 months, and (ii) setting excess mortality to 0 across all age groups. For GBD 2021, the Healthcare Access and Quality (HAQ) Index was the only country-level covariate used to assess excess mortality.

Mortality estimates were generated using the Cause of Death Ensemble Model (CODEm). CODEm systematically evaluates various statistical modeling techniques and covariate combinations for predictive accuracy, integrating these outcomes to produce robust cause-specific mortality estimates. Covariates integrated within CODEm for estimating the fatal burden of AYA MM included the HAQ Index, log-transformed lag-distributed income per capita, and the Sociodemographic Index (SDI). Alternative modeling strategies were employed for causes with sparse data, significant reporting variations over the analysis period, or epidemiological irregularities.

To assess temporal trends in age-specific rates, the estimated Annual Percentage Change (APC) was computed. APC values were calculated through the regression model:

$$y\, = \,\alpha \, + \,\beta x\, + \,\varepsilon$$

in which ‘y’ denotes the natural logarithm of the AsR, and ‘x’ signifies the calendar year.

The resulting APC was determined by applying the formula: 100 × (exp(β) − 1).

Positive APC values with corresponding 95% uncertainty intervals (UI) reflect an upward trend in AsR, whereas negative APC values and their 95% UIs indicate a downward trend. All temporal trend estimates, including annual percentage change (APC), were obtained directly from the Global Burden of Disease 2021 analytical framework; no independent joinpoint or post-hoc trend modeling was performed by the authors.

We computed two sample t-test to get the differences between the AsR of 1990 and 2021. The process is to estimate the standard deviations for each group, which is plugged into the denominator to compute the statistic. All the statistical analysis was automated using R (version 4.4.1, Race for Your Life)[15].

For projection to 2040, we applied a logarithmic transformation to the time series data to reduce volatility. Both the Auto-Regressive Integrated Moving Average (ARIMA) model and the Exponential Smoothing State Space (ETS) model were utilized to forecast incidence rates and death rates. For the ARIMA model, the best-performing version was automatically selected based on the minimum values of AIC, AICc, or BIC. Similarly, for ETS modeling, the best-performing model was chosen using the same criteria (AIC, AICc, or BIC). Model performance was evaluated by comparing the Root Mean Square Error (RMSE) and Mean Absolute Error (MAE) of the in-sample model fits from 1990 to 2021. The optimal model, based on these metrics, was then used to perform out-of-sample forecasting for the period from 2022 to 2040.

## Result

### Global

In 2021, global multiple myeloma (MM) incidence among AYA (15–39 yrs) was 2,536 (95% UI 1909, 2973) with incidence rate (IR) of 0.09 (95% UI 0.06, 0.10) per 100,000 patient-years, and 1,579 deaths (95% UI: 1176, 1871) with death rate (DR) of 0.05 (95% UI 0.04, 0.06) per 100,000 patient-years. (Table 1, Supplemental Fig. S2).

**Table 1:** The Incidence Rate (IR), Death Rate (DR) in 1990 and 2021 and Annual percentage changes (APC) of incidence rate (IR) and death rate (DR) from 1990 to 2021 of adolescent and young adult (AYA) multiple myeloma in global, Socio-demographic Index (SDI) regions, and 21 Global Burden of Disease (GBD) regions per 100,000 population for both sexes combined. 95% UI 95% uncertainty interval

Location	1990 IR (95% UI)	2021 IR (95% UI)	p-value	APC of IR (95% UI)	1990 DR (95% UI)	2021 DR (95% UI)	p-value	APC of DR (95% UI)
Global	0.05 (0.04, 0.05)	0.09 (0.06, 0.10)	< 0.001	0.84 (0.44, 1.22)	0.03 (0.03, 0.04)	0.05 (0.04, 0.06)	0.002	0.60 (0.24, 0.94)
High SDI	0.09 (0.09, 0.10)	0.12 (0.11, 0.13)	< 0.001	0.32 (0.18, 0.45)	0.06 (0.05, 0.06)	0.06 (0.05, 0.06)	NA	0.05 (− 0.08, 0.14)
High-middle SDI	0.06 (0.05, 0.07)	0.15 (0.10, 0.19)	< 0.001	1.50 (0.74, 2.21)	0.04 (0.04, 0.05)	0.08 (0.06, 0.10)	< 0.001	0.93 (0.31, 1.45)
Middle SDI	0.03 (0.03, 0.05)	0.09 (0.07, 0.11)	< 0.001	1.73 (0.87, 2.51)	0.03 (0.02, 0.04)	0.06 (0.04, 0.07)	< 0.001	1.24 (0.54, 1.88)
Low-middle SDI	0.03 (0.02, 0.04)	0.05 (0.04, 0.08)	0.10	0.98 (0.49, 2.13)	0.02 (0.02, 0.03)	0.04 (0.03, 0.06)	0.03	0.81 (0.35, 1.85)
Low SDI	0.02 (0.01, 0.04)	0.04 (0.02, 0.05)	0.03	0.47 (0.02, 1.71)	0.02 (0.01, 0.03)	0.03 (0.02, 0.04)	0.08	0.39 (− 0.03, 1.54)
Andean Latin America	0.05 (0.04, 0.07)	0.09 (0.06, 0.13)	0.03	0.70 (0.11, 1.37)	0.04 (0.03, 0.05)	0.06 (0.04, 0.08)	0.03	0.37 (− 0.08, 0.88)
Australasia	0.13 (0.11, 0.15)	0.16 (0.12, 0.22)	0.17	0.30 (− 0.08, 0.88)	0.06 (0.05, 0.06)	0.05 (0.04, 0.06)	0.02	− 0.15 (− 0.32, 0.06)
Caribbean	0.09 (0.07, 0.11)	0.10 (0.08, 0.12)	0.24	0.13 (− 0.13, 0.42)	0.05 (0.05, 0.06)	0.06 (0.05, 0.07)	0.08	0.04 (− 0.15, 0.25)
Central Asia	0.06 (0.05, 0.07)	0.10 (0.08, 0.11)	< 0.001	0.72 (0.38, 1.11)	0.05 (0.04, 0.05)	0.07 (0.06, 0.08)	< 0.001	0.60 (0.30, 0.97)
Central Europe	0.07 (0.06, 0.07)	0.09 (0.08, 0.10)	< 0.001	0.34 (0.19, 0.56)	0.05 (0.05, 0.05)	0.05 (0.05, 0.06)	0.5	0.11 (-0.01, 0.22)
Central Latin America	0.06 (0.06, 0.07)	0.10 (0.09, 0.12)	< 0.001	0.66 (0.47, 0.89)	0.05 (0.05, 0.05)	0.07 (0.06, 0.07)	0.0	0.39 (0.23, 0.54)
Central Sub-Saharan Africa	0.01 (0.01, 0.02)	0.02 (0.01, 0.02)	0.02	0.20 (-0.27, 0.86)	0.01 (0.01, 0.02)	0.01 (0.01, 0.02)	0.5	0.14 (− 0.31, 0.78)
East Asia	0.03 (0.02, 0.05)	0.16 (0.09, 0.22)	< 0.001	4.72 (1.34, 8.45)	0.02 (0.02, 0.04)	0.09 (0.05, 0.12)	< 0.001	3.10 (0.68, 5.75)
Eastern Europe	0.08 (0.07, 0.09)	0.13 (0.12, 0.14)	< 0.001	0.65 (0.44, 0.91)	0.05 (0.05, 0.06)	0.07 (0.07, 0.08)	0.002	0.38 (0.22, 0.57)
Eastern Sub-Saharan Africa	0.04 (0.02, 0.05)	0.06 (0.03, 0.08)	0.03	0.64 (0.06, 2.00)	0.03 (0.01, 0.04)	0.05 (0.03, 0.07)	0.03	0.55 (− 0.01, 1.81)
High-income Asia Pacific	0.05 (0.04, 0.06)	0.06 (0.05, 0.08)	0.19	0.22 (− 0.07, 0.63)	0.03 (0.03, 0.03)	0.03 (0.02, 0.03)	NA	− 0.15 (− 0.27, 0.04)
High-income North America	0.10 (0.09, 0.10)	0.09 (0.08, 0.10)	0.02	− 0.08 (− 0.16, 0.02)	0.07 (0.07, 0.07)	0.05 (0.05, 0.06)	< 0.001	− 0.21 (−0.25, −0.17)
North Africa and Middle East	0.05 (0.03, 0.07)	0.08 (0.06, 0.11)	0.05	0.77 (0.10, 1.66)	0.04 (0.02, 0.05)	0.05 (0.04, 0.07)	0.19	0.39 (−0.09, 1.08)
Oceania	0.03 (0.01, 0.04)	0.03 (0.02, 0.05)	0.5	0.32 (−0.06, 0.96)	0.02 (0.01, 0.03)	0.03 (0.01, 0.04)	0.08	0.30 (−0.09, 0.96)
South Asia	0.03 (0.02, 0.04)	0.06 (0.05, 0.09)	0.03	0.89 (0.37, 2.49)	0.03 (0.02, 0.04)	0.05 (0.03, 0.07)	0.03	0.69 (0.24, 2.14)
Southeast Asia	0.02 (0.01, 0.02)	0.03 (0.02, 0.05)	0.16	0.91 (0.46, 1.68)	0.01 (0.01, 0.02)	0.02 (0.02, 0.03)	0.08	0.70 (0.31, 1.38)
Southern Latin America	0.08 (0.07, 0.09)	0.10 (0.08, 0.12)	0.03	0.24 (0.01, 0.55)	0.06 (0.05, 0.07)	0.06 (0.05, 0.06)	0.5	− 0.02 (−0.17, 0.17)
Southern Sub-Saharan Africa	0.10 (0.07, 0.13)	0.16 (0.10, 0.22)	0.03	0.55 (0.05, 1.21)	0.08 (0.05, 0.10)	0.12 (0.08, 0.16)	0.03	0.47 (0.00, 1.06)
Tropical Latin America	0.06 (0.06, 0.06)	0.12 (0.11, 0.13)	0.00	0.97 (0.80, 1.16)	0.05 (0.04, 0.05)	0.08 (0.07, 0.08)	0.00	0.68 (0.57, 0.79)
Western Europe	0.12 (0.11, 0.13)	0.15 (0.14, 0.17)	0.004	0.23 (0.09, 0.41)	0.06 (0.06, 0.06)	0.05 (0.05, 0.05)	0.00	− 0.16 (−0.22, −0.11)
Western Sub-Saharan Africa	0.01 (0.00, 0.01)	0.02 (0.01, 0.03)	0.02	0.81 (0.08, 1.70)	0.01 (0.00, 0.01)	0.01 (0.00, 0.02)	0.50	0.69 (0.01, 1.57)

**Fig. 1:**
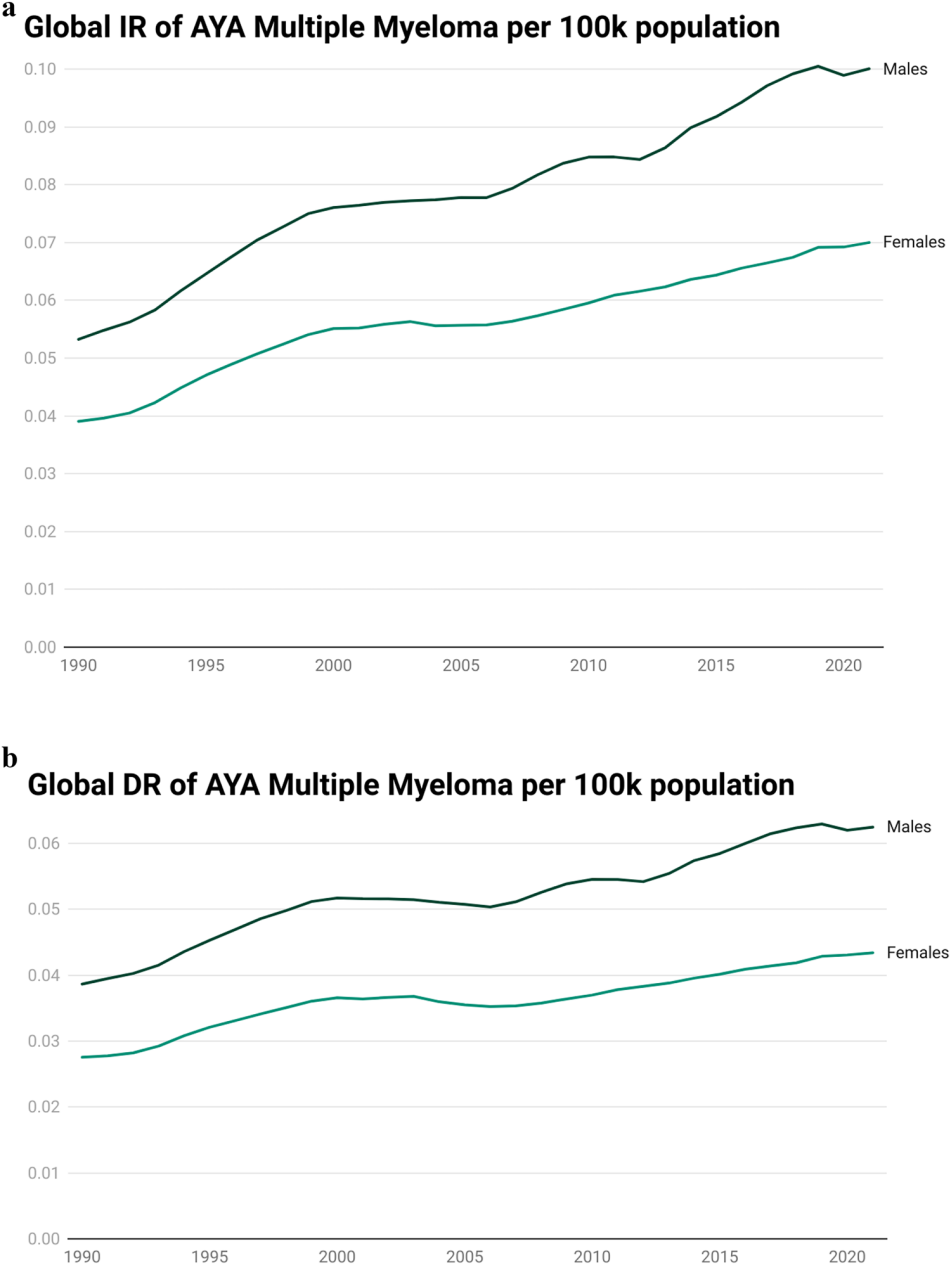
Trends of incidence rate (IR) (Fig. 1A), death rate (DR) (Fig. 1B), of adolescent and young adult (AYA) multiple myeloma per 100,000 patient-years from 1990 to 2021

From 1990 to 2021, IR and DR increased (annual percentage change (APC) of 0.84 for IR and 0.60 for DR). (Table 1 and Figs. 1, 3).

### By Sex

**Fig. 2:**
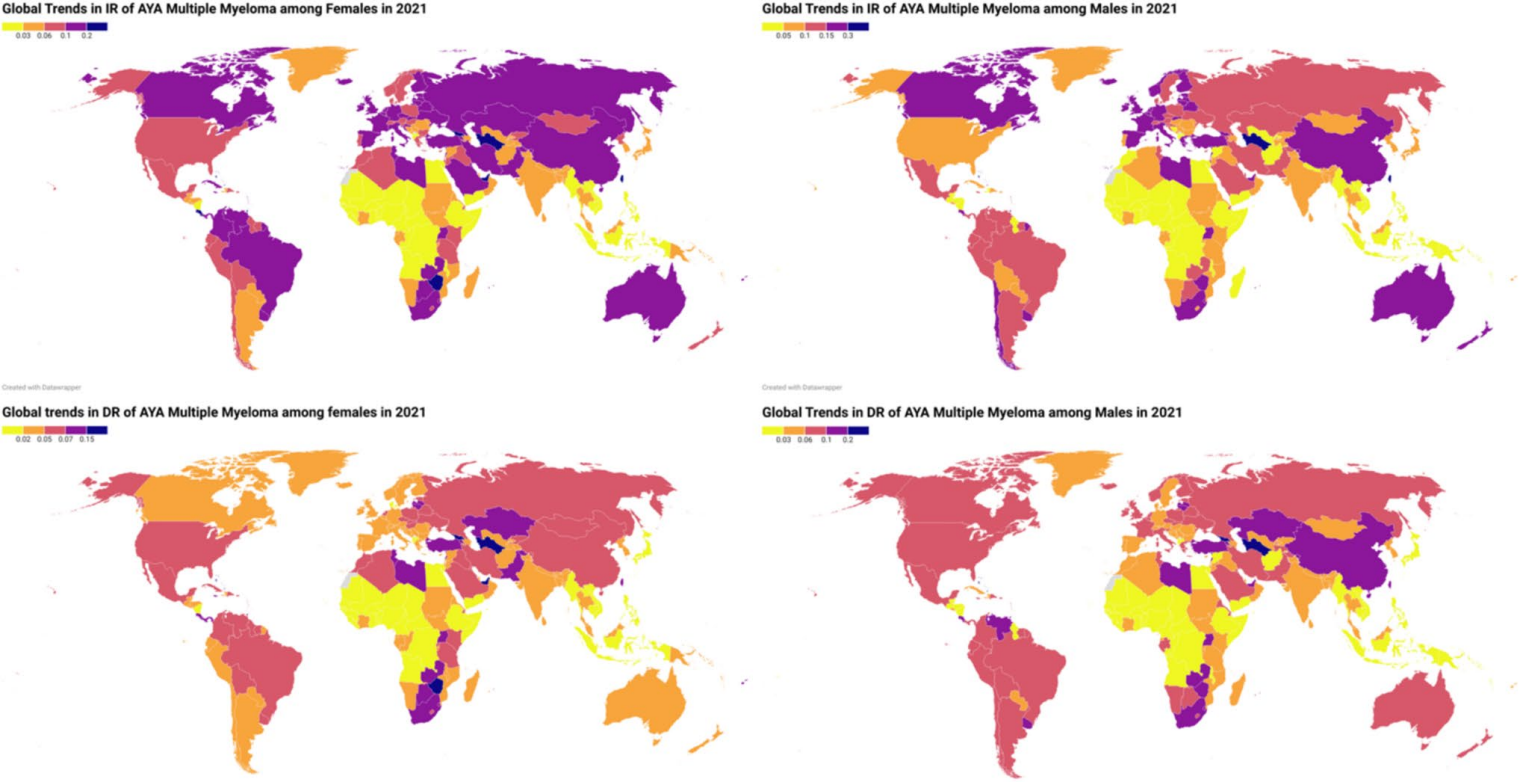
Global trends of incidence rates (IR) and death rates (DR) of adolescent and young adult (AYA) Multiple myeloma among males and females in 2021 per 100,000 patient-years

**Fig. 3:**
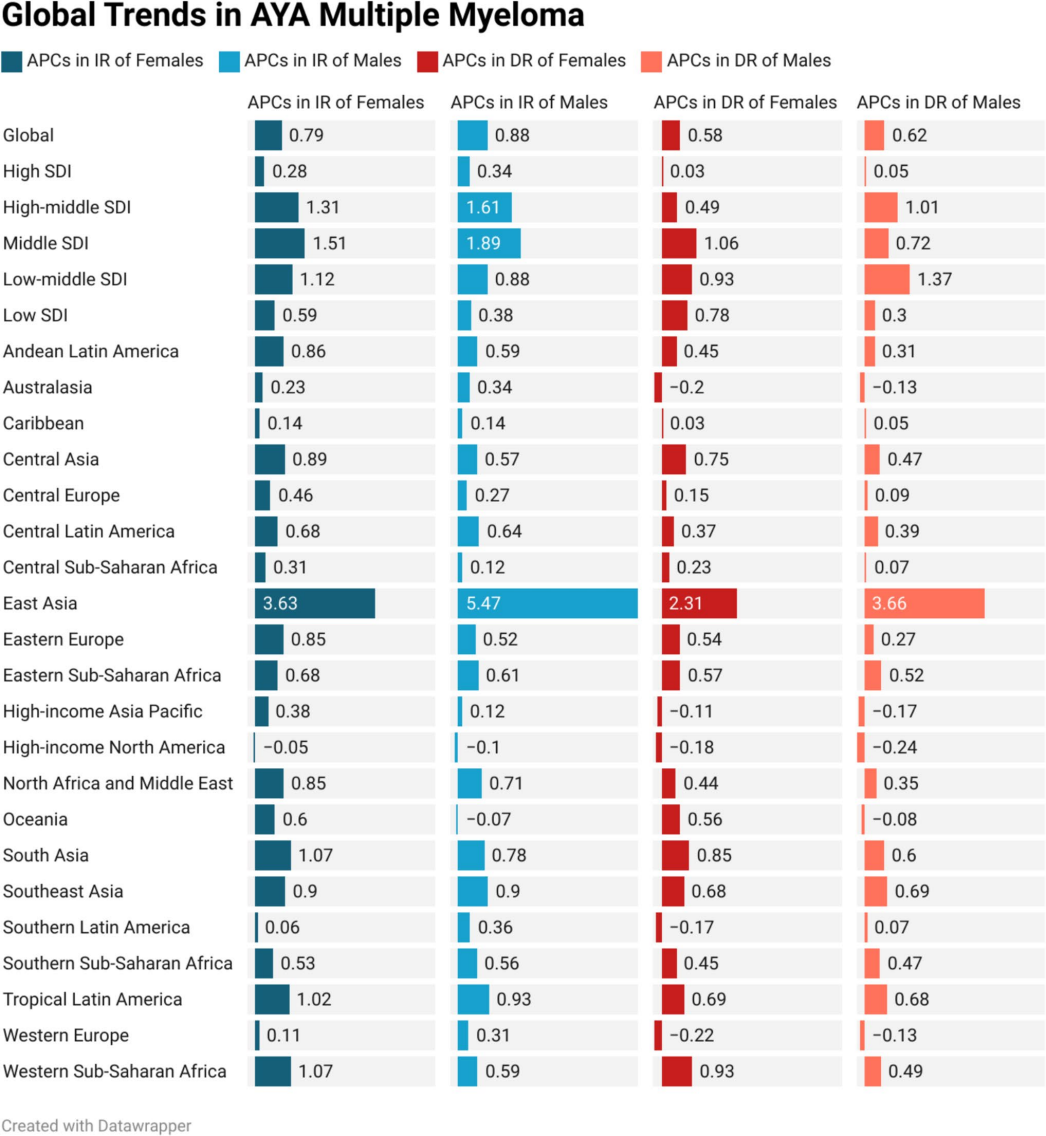
Trends of Annual percentage changes (APC) of incidence rates (IR) and death rates (DR) of adolescent and young adult (AYA) Multiple myeloma from 1990 to 2021 in Global, Socio-demographic Index (SDI) regions, and 21 Global Burden of Disease (GBD) regions per 100,000 population

**Fig. 4:**
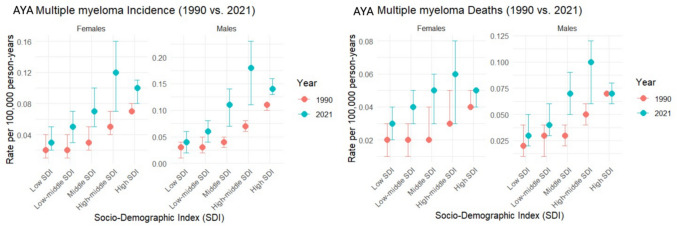
The Incidence Rate (IR), Death Rate (DR) of adolescent and young adult (AYA) Multiple myeloma in 1990 and 2021 across Socio-Demographic Index (SDI) regions per 100,000

In 2021, the global incidence of multiple myeloma among adolescents and young adults (AYA) aged 15–39 years was 1510 cases (95% UI 1073, 1786) in males and 1,025 cases (95% UI 673, 1292) in females, while the number of deaths attributed to multiple myeloma in the same age group was 943 (95% UI 671, 1101) for males and 636 (95% UI 415, 798) for females. Supplementary Table S1, Fig. S1, revealed the global trends of AYA MM in males and females. Figure 2 and Supplemental Fig. S1 represent the country-wise IR and DR and annual percentage change (APC) of IR and DR through world maps. Table 1 and Figs. 3, 4 revealed changed of IR and DR in global, SDI regions, and 21 GBD regions.

### By SDI regions

Table 1, supplementary Table S1, Fig. S3, and supplemental Fig. S2 revealed a comparative analysis of the IR, DR, and APC of IR, DR by males, females, and both sexes in 5 SDI regions. Figure 4 compared 1990 and 2021 IR and DR in SDI regions.

In 2021, global MM IR and DR among AYAs varied across SDI regions. The high middle SDI had the highest IR (0.15, 95% UI 0.10, 0.19) and DR (0.08, 95% UI 0.06, 0.10), while the low SDI had the lowest IR (0.04, 95% UI 0.02, 0.05) and DR (0.03, 95% UI 0.02, 0.04).

From 1990 to 2021, global MM IR and DR among AYAs (15–39 yrs) varied across SDI regions. The middle SDI had the highest increase of IR (APC: 1.73; 95% UI 0.87, 2.51) and DR (1.24; 95% UI 0.54–1.88), while the high SDI had the lowest increase of IR (0.32; 95% UI 0.18, 0.45) and DR (0.05; 95% UI − 0.08, 0.14).

### By GBD regions

In 2021, regional MM IR and DR among AYAs (15, 39 yrs) varied across regions. The highest IRs were observed in East Asia (0.16, 95% UI 0.09, 0.22), Australasia (0.16, 95% UI 0.12, 0.22), and Southern Sub-Saharan Africa (0.16, 95% UI 0.10, 0.22), while the lowest IRs were in Western Sub-Saharan Africa (0.02, 95% UI 0.01, 0.03), Central Sub-Saharan Africa (0.02, 95% UI 0.01, 0.02), and Southeast Asia (0.03, 95% UI 0.02, 0.05). The highest DRs were recorded in Southern Sub-Saharan Africa (0.12, 95% UI 0.08, 0.16), East Asia (0.09, 95% UI 0.05, 0.12), and Tropical Latin America (0.08, 95% UI 0.07, 0.08), whereas the lowest DRs were in Western Sub-Saharan Africa (0.01, 95% UI 0.00, 0.02), Central Sub-Saharan Africa (0.01, 95% UI 0.01, 0.02), and Southeast Asia (0.02, 95% UI 0.02, 0.03).

From 1990 to 2021, regional trends in MM IR and DR among AYAs (15, 39 yrs) exhibited substantial variation. The highest IR increases were observed in East Asia (4.72, 95% UI 1.34, 8.45), Tropical Latin America (0.97, 95% UI 0.80, 1.16). High-income North America (− 0.08, 95% UI − 0.16, 0.02) showed a decline in IR. For DR, the highest increases were reported in East Asia (3.10, 95% UI 0.68, 5.75) and Southeast Asia (0.70, 95% UI 0.31, 1.38). High-income North America (− 0.21, 95% UI − 0.25, − 0.17), Western Europe (− 0.16, 95% UI − 0.22, − 0.11), Australasia (− 0.15, 95% UI − 0.32, 0.06), High-income Asia Pacific (− 0.15, 95% UI − 0.27, 0.04), and the Caribbean (0.04, 95% UI − 0.15, 0.25) showed a declining pattern of death rates.

Table 1, supplementary Table S1, Fig. S3, and supplemental Fig. S2 revealed a comparative analysis of the IR, DR, and APC of IR, DR by males, females, and both sexes in 21 GBD regions.

### By 204 countries

In 2021, the highest MM IR and DR among AYAs (15–39 yrs) was observed in Mauritius [IR of 7.30 (95% UI 5.77, 9.26), and DR of 6.44 (95% UI 5.58, 7.31)].

From 1990 to 2021, the highest increase in IR and DR was seen in Mauritius, with the APC of IR of 7.30 (95% UI 5.77, 9.26), and APC of DR of 6.44 (95% UI 5.58, 7.31). The highest decrease in IR was shown to be in Greenland with an APC of − 0.42 (95% UI − 0.70, 0.28). Finland [APC: − 0.55 (95% UI − 0.63, − 0.43)] and Switzerland [APC: − 0.54 (95% UI − 0.62, − 0.45)] showed the highest decrease in Incidence rates.

Figure 2 and Supplemental Fig. S1 represent the country-wise IR and DR and annual percentage change (APC) of IR and DR through world maps.

By risk factors:

In 2021, the percentage of MM deaths attributable to high body-mass index (BMI) among AYAs (15–39 yrs) globally was 6.52% (95% UI: − 2.30, 16.52) in females and 5.97% (95% UI − 1.98, 15.04) in males. From 1990 to 2021, the percentage of MM deaths attributable to high BMI among AYAs (15–39 yrs) globally increased by 47.58% (95% UI 34.97, 87.05) in males and by 45.74% (95% UI 33.04, 96.71) in females.

**Fig. 5:**
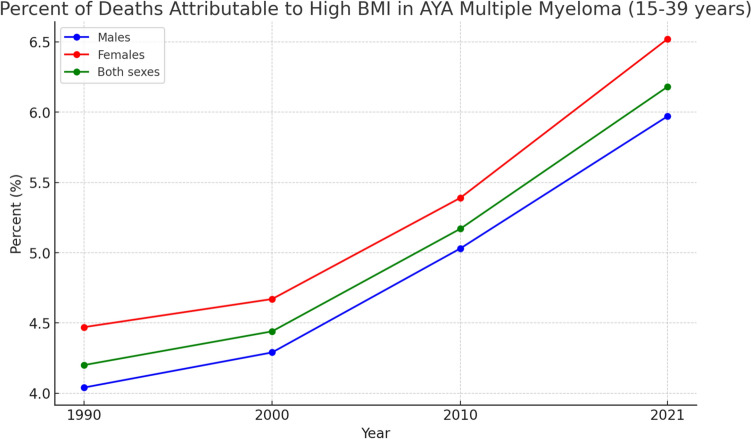
Trends in the impact of risk factor (High BMI) on adolescent and young adult (AYA) Multiple myeloma-related deaths from 1990 to 2021

Figure 5 revealed the trends in percent of deaths attributed to high BMI in AYA MM from 1990 to 2021. Supplemental Fig. S3and Fig. S4 demonstrate the percentage of AYA MM deaths associated with high BMI in 2021, along with the APC from 1990 to 2021.

## Projection

**Fig. 6:**
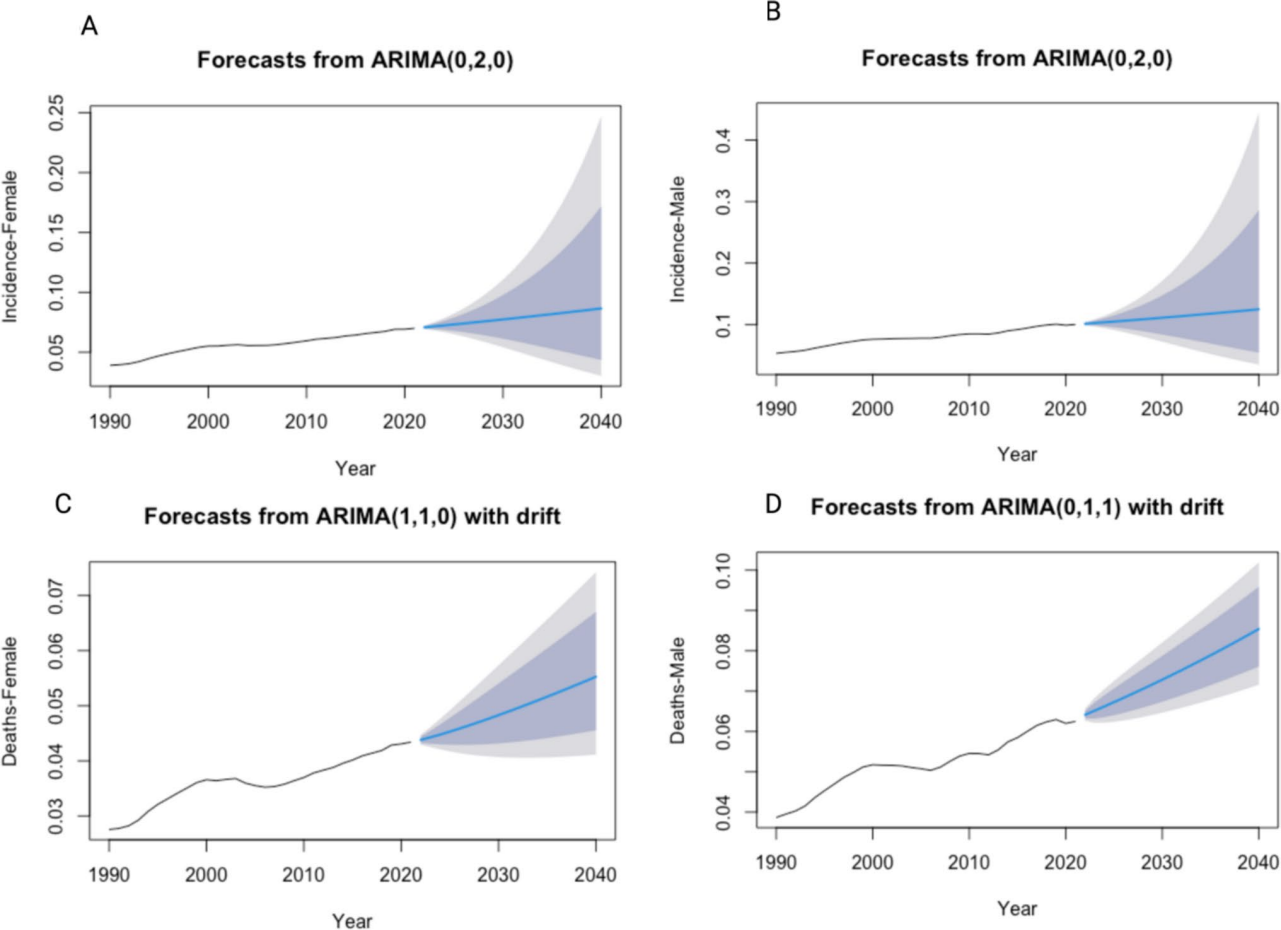
Projection of IR (Incidence Rate) (6 A, B), DR (Death Rate)(6 C, D) of adolescent and young adult (AYA) Multiple Myeloma from 2022 to 2040 per 100,000 patient-years

Figure 6(A, B, C, D) illustrates the projection of IR and DR among males and females till 2040. Both the IR and DR of males and females revealed increasing trends till 2040

## Discussion

Multiple myeloma (MM) is rare in adolescents and young adults (AYAs), representing less than 2% of all MM cases. The clinical presentation in AYAs is generally similar to that of older patients, with common features including anemia, renal impairment, and hypercalcemia. However, AYAs may have a higher incidence of high-risk cytogenetic abnormalities, such as del(17p) and t (4;14), which are associated with poorer outcomes [16, 17].

In 2021, AYAs (15–39 years) accounted for 1.71% of global MM cases and 1.36% of global MM deaths. Comparatively, in 1990, AYAs accounted for 1.82% of global MM cases and 1.53% of global MM deaths. Several factors have driven the increasing incidence of MM among AYAs. Firstly, improved diagnostic capabilities and awareness, including the widespread use of serum protein electrophoresis and advanced imaging techniques, have enabled earlier detection of MM [16] Secondly, advancements in treatment modalities, including novel agents such as proteasome inhibitors (e.g., bortezomib), immunomodulatory drugs (e.g., lenalidomide), and monoclonal antibodies (e.g., daratumumab), have significantly improved patient outcomes. For example, the combination of bortezomib, lenalidomide, and dexamethasone (VRd) has been shown to enhance OS rates significantly [18, 19] In a systematic review, Tanguay et al. noted that young patients receiving VRd demonstrated improved outcomes compared to older cohorts, underscoring the potential benefits of tailored treatment strategies for AYAs [20] Thirdly, the global rise in lifestyle and metabolic risk factors, particularly among younger populations, has further contributed to the growing incidence of MM.

Globally, the incidence and mortality of AYA MM increased from 1990 to 2021, with incidence rates (IR) rising faster than death rates (DR). Despite the aggressive nature of MM in some AYAs, the overall prognosis can be favorable with modern therapies. AYAs often tolerate more intensive therapies, including high-dose chemotherapy followed by autologous stem cell transplantation (ASCT), due to fewer comorbidities. Studies have shown that AYAs achieve significant survival benefits from ASCT, with median overall survival (OS) extending beyond 10 years in some cohorts. For instance, the 5-year OS rate for AYAs can be as high as 71–84%, with the Japanese cohort reporting a 5-year OS of 71% and the French cohort reporting 84%. Additionally, the median OS in the French study is 14.5 years, which is longer than that typically observed in older patients. This is a marked improvement compared to the general MM population, where the five-year survival rate has increased from approximately 27% in the late 1980s to around 49% between 2005 and 2011. [21] Although advances in multiple myeloma therapy have led to substantial improvements in survival, the increasing mortality observed in this study should be interpreted in the context of rising incidence, population growth, and persistent global inequities in healthcare access. Improved diagnostic capacity and death reporting likely contribute to observed increases in multiple myeloma incidence and mortality trends, though the specific magnitude of this effect is difficult to quantify and varies substantially by region and income level. Importantly, multiple myeloma remains an incurable disease, and prolonged survival does not eliminate cumulative mortality risk, particularly in regions where access to autologous stem cell transplantation and novel agents is limited. [22–24] Consequently, population-level mortality trends may continue to rise despite meaningful improvements in individual-level outcomes.

Males were more affected than females, experiencing higher incidence and mortality rates, with faster increases over time compared to females from 1990 to 2021. Several factors contribute to this disparity, including biological differences (e.g., higher hyperdiploidy prevalence) [16], greater exposure to risk factors such as obesity, inactivity, and a higher prevalence of MGUS [16, 25], delayed healthcare access [26], socioeconomic disparities (occupational exposures, lifestyle) [26], and genetic predispositions. [27].

Among different sociodemographic index (SDI) regions, high-middle SDI regions exhibited the highest IR and DR, while low SDI regions had the lowest. Over time, middle SDI regions experienced the greatest increases in IR and DR, whereas high SDI regions had the lowest increases. Since 2021, Southern Sub-Saharan Africa reported the highest death rate, while Western and Central Sub-Saharan Africa had the lowest DR. East Asia and Tropical Latin America have shown rising MM incidence from 1990 to 2021. In contrast, North America and Western Europe experienced a declining incidence. Mauritius has the highest IR and DR, while Greenland, Finland, and Switzerland experienced declining IR and DR. These regional differences could be due to healthcare access, environmental factors, and genetics. However, underreporting and limited diagnostics could also contribute to the low IR and DR.

Notably, obesity is a key modifiable risk factor associated with MM. In 2021, approximately 6.52% of MM deaths in AYAs were attributable to high body mass index (BMI), reflecting a notable increase from 1990 to 2021. This trend highlights the importance of addressing lifestyle factors in prevention strategies for this demographic. Additionally, type 2 diabetes mellitus (T2DM) has been linked to an increased risk of MM, suggesting that metabolic disorders play a critical role in MM pathogenesis [28] Issa et al., 2011). Although genetic, environmental, and lifestyle factors are important contributors to multiple myeloma risk, quantitative assessment of these exposures was not feasible in this study because high body-mass index is the only risk factor for multiple myeloma mortality currently available within the GBD framework.

While the study suggests an increasing trend in IR and DR from 1990 to 2040 for AYA MM, this study has several limitations that warrant consideration. First, the reliance on modeled data from the Global Burden of Disease (GBD) database introduces potential inaccuracies due to underreporting, limited availability of country-specific data, and reliance on predictive modeling based on data patterns from neighboring regions. [14] These issues may affect the precision of incidence and mortality estimates. Second, the analysis did not stratify MM by subtypes (e.g., Smoldering MM), which limits the ability to assess trends and risk factors specific to these high-burden and clinically distinct categories. Third, variations in data quality and diagnostic accuracy across the 30-year study period (1990–2021) and between regions could have influenced the observed trends. These discrepancies are particularly pronounced in low- and middle-SDI regions, where healthcare infrastructure, diagnostic facilities, and data collection systems are less developed. Fourth, mortality estimates reflect deaths occurring within the 15–39-year age group in a given year and do not capture delayed mortality among individuals diagnosed with multiple myeloma in adolescence or young adulthood who die at older ages, due to the absence of longitudinal, patient-level linkage in the GBD framework. Finally, the projections to 2040 rely on historical trends and assume that current interventions will remain consistent. Unforeseen factors such as the emergence of new risk factors, sociopolitical instability, or breakthroughs in cancer prevention and treatment could alter the trajectory of AYA MM burden. Future research should prioritize real-time data integration, enhanced stratification by clinical subtypes, and evaluation of evolving risk factors to address these gaps.

## Supplementary Information

Below is the link to the electronic supplementary material.

Supplementary file1 (DOCX 1812 KB)

## Data Availability

No datasets were generated or analysed during the current study.
