# Supplementary material for: Multiple myeloma in adolescents and young adults(AYA): global epidemiological trends, risk factors, and future projections (1990–2040)
Source: Cancer Causes Control. 2026 Feb 19;37(3):51. doi: 10.1007/s10552-026-02142-3 (PMC12920330; doi:10.1007/s10552-026-02142-3)
Supplement: Supplementary file 1 — Supplementary file1 (DOCX 1812 KB) [file 10552_2026_2142_MOESM1_ESM.docx]

Supplemental Figures:

Supplemental Figure 1: Global trends in the annual percentage change (APC) of incidence rates (IR) and death rates (DR) of adolescent and young adult (AYA) multiple myeloma among males and females from 1990 to 2021 per 100,000 patient-years: Sup Fig: 1A: Female IR, 1B: Male IR, 1C: Female DR, 1D: Male DR.

Sup Fig 1A:


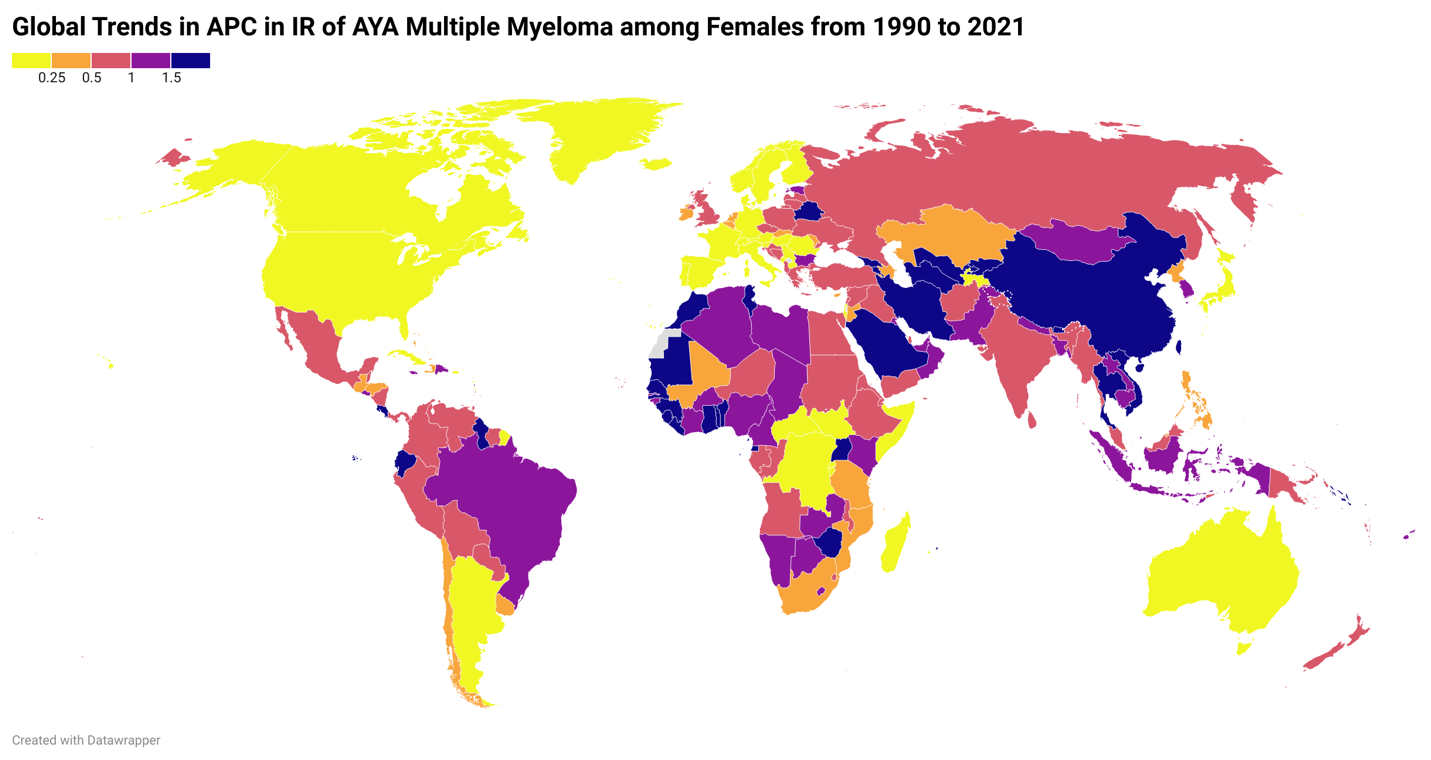


Sup Fig 1B:


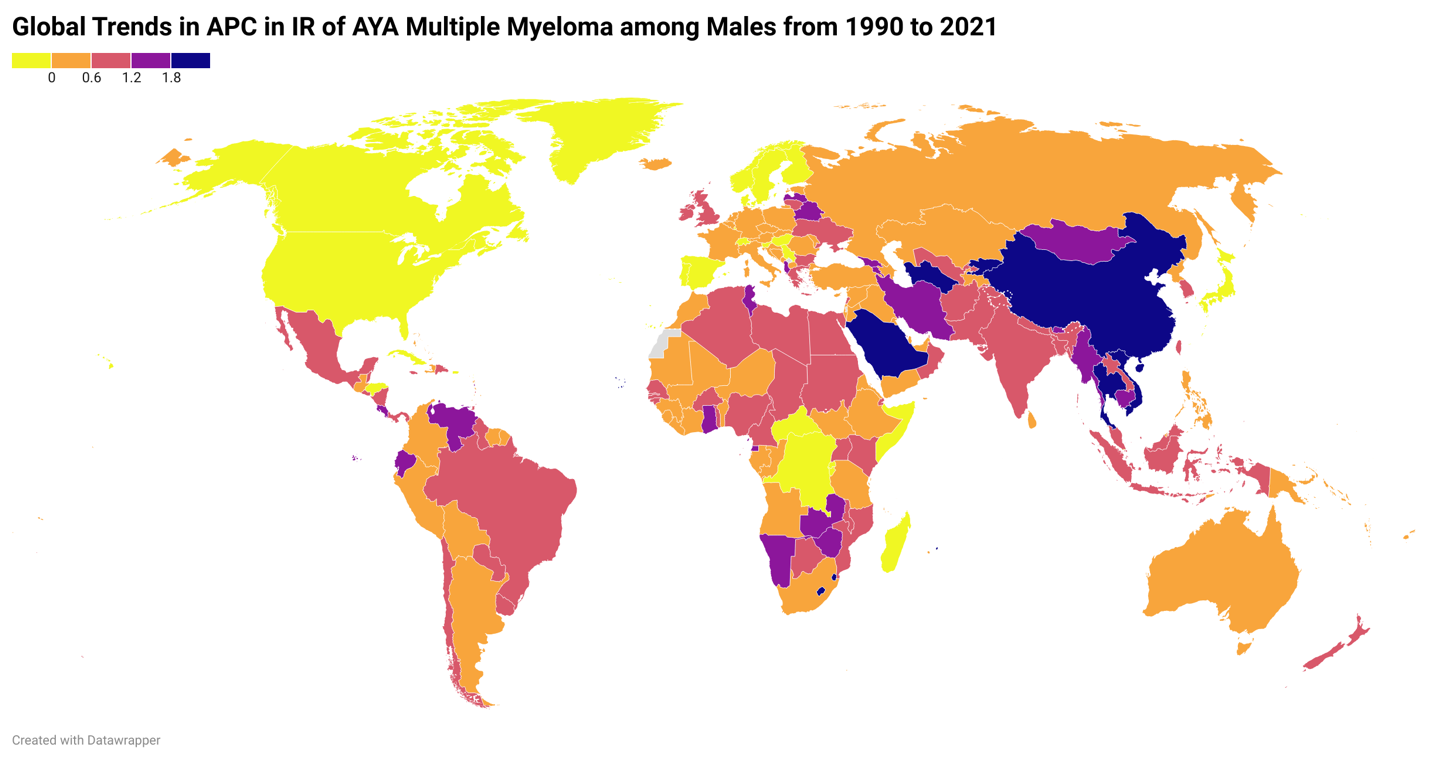


Sup Fig 1C:


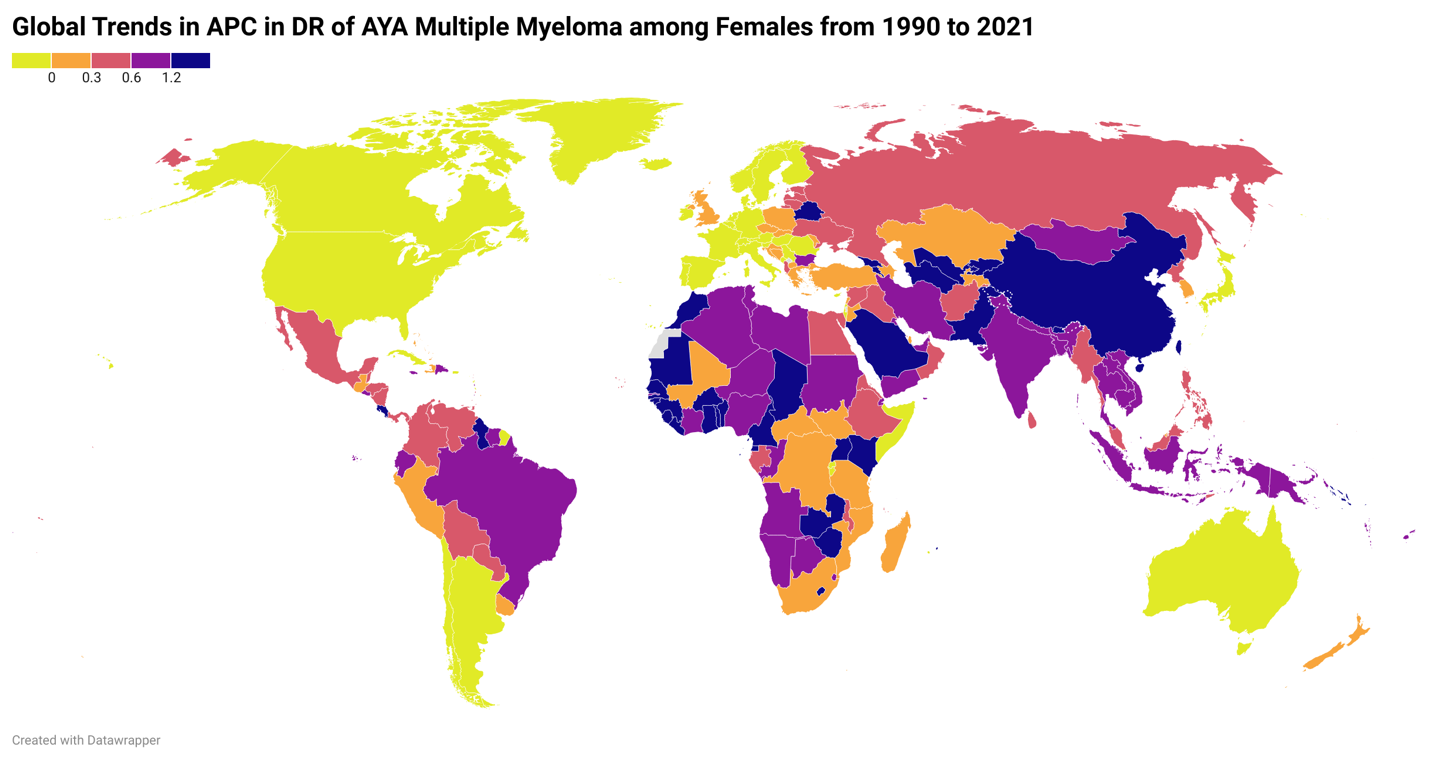


Sup Fig 1D:


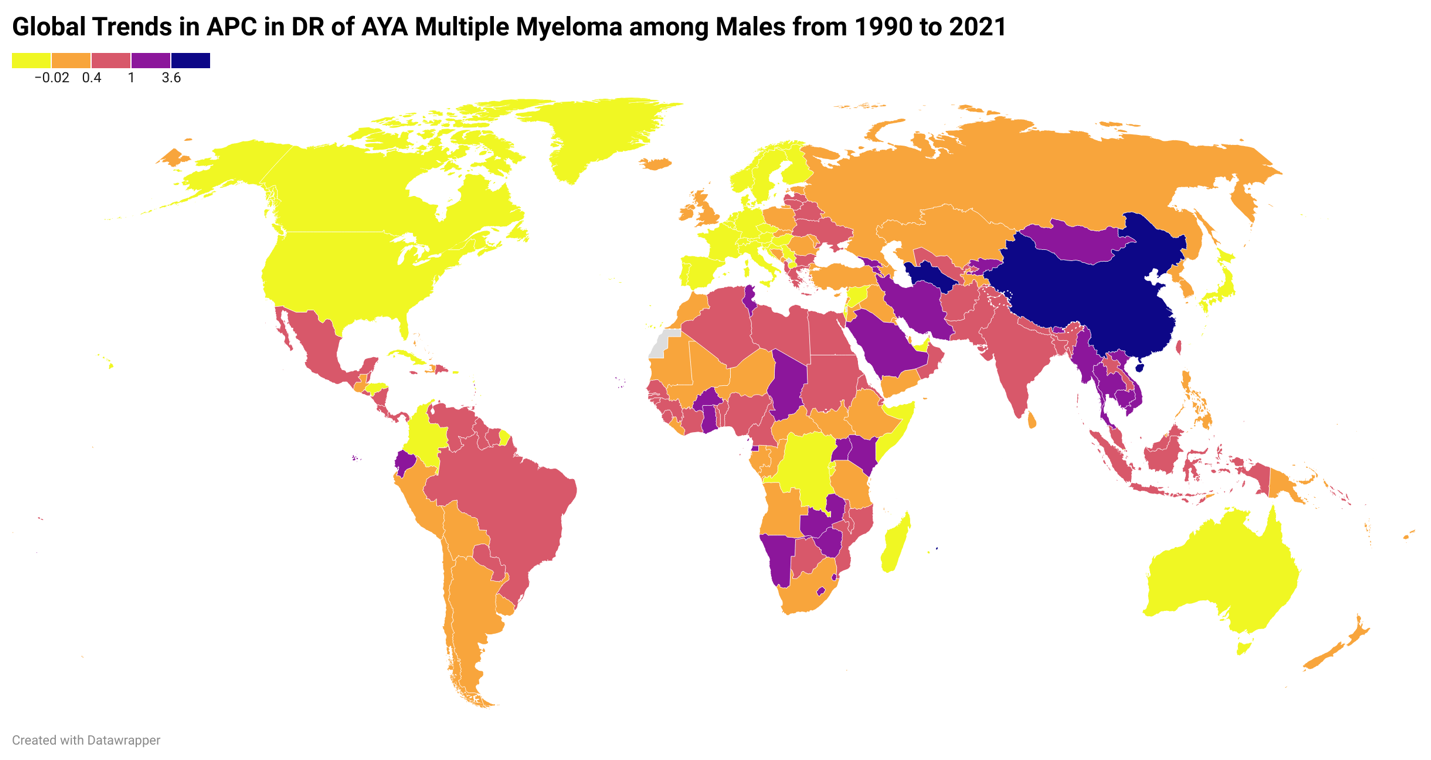


Supplemental Figure 2: in 2021, the incidence and death rates of adolescent and young adult (AYA) multiple myeloma in Global, Socio-demographic Index (SDI) regions, and 21 Global Burden of Disease (GBD) regions per 100,000 population among males, females and both sexes together.


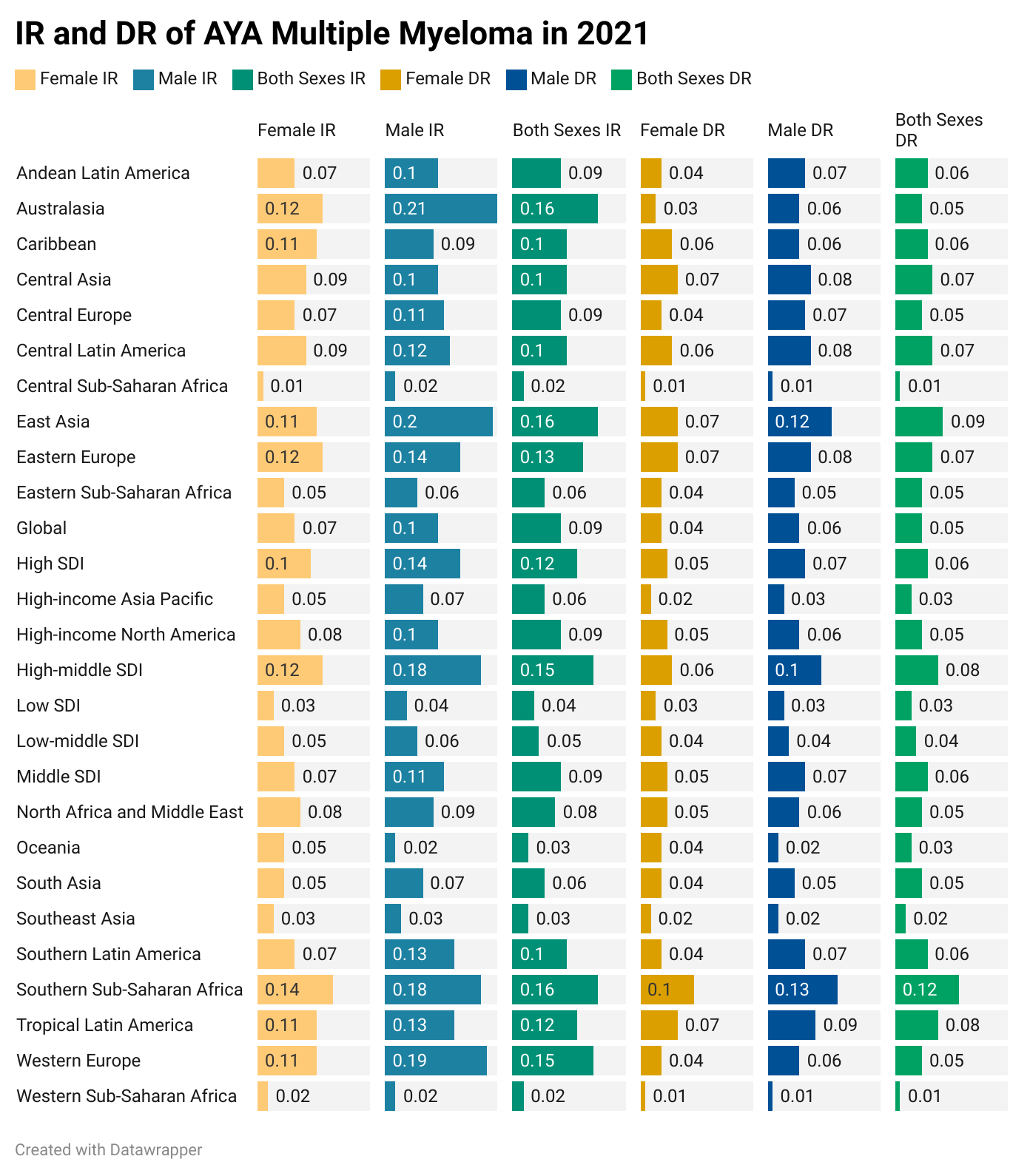


Supplemental Figure 3: In 2021, the percent of deaths of adolescent and young adult (AYA) multiple myeloma attributed to high Body-mass-index (BMI) among males, females and both sexes together.


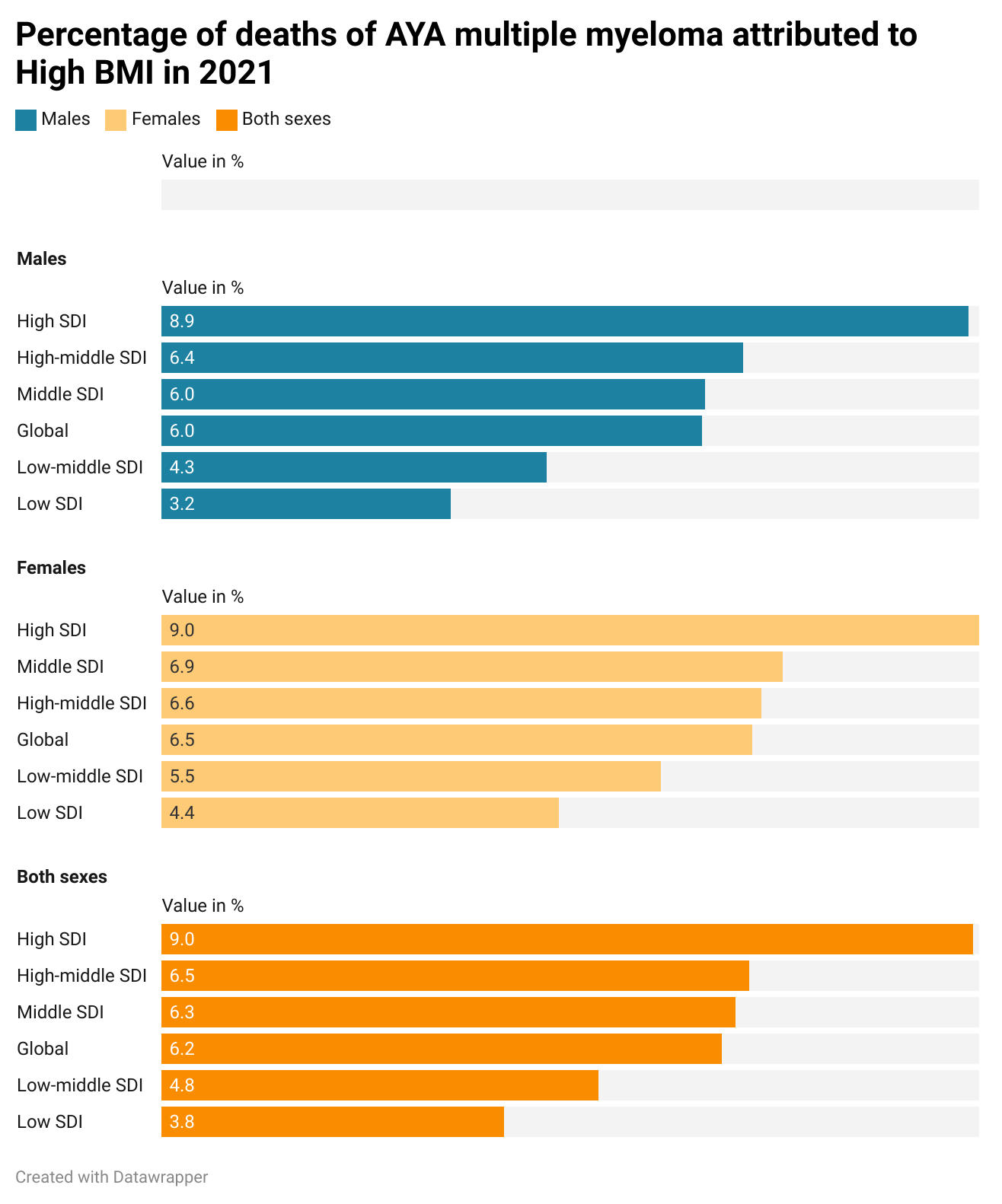


Supplemental Figure 4: From 1990 to 2021, the annual percentage changes (APC) of deaths of adolescent and young adult (AYA) multiple myeloma attributed to high Body-mass-index (BMI) among males, females and both sexes together.


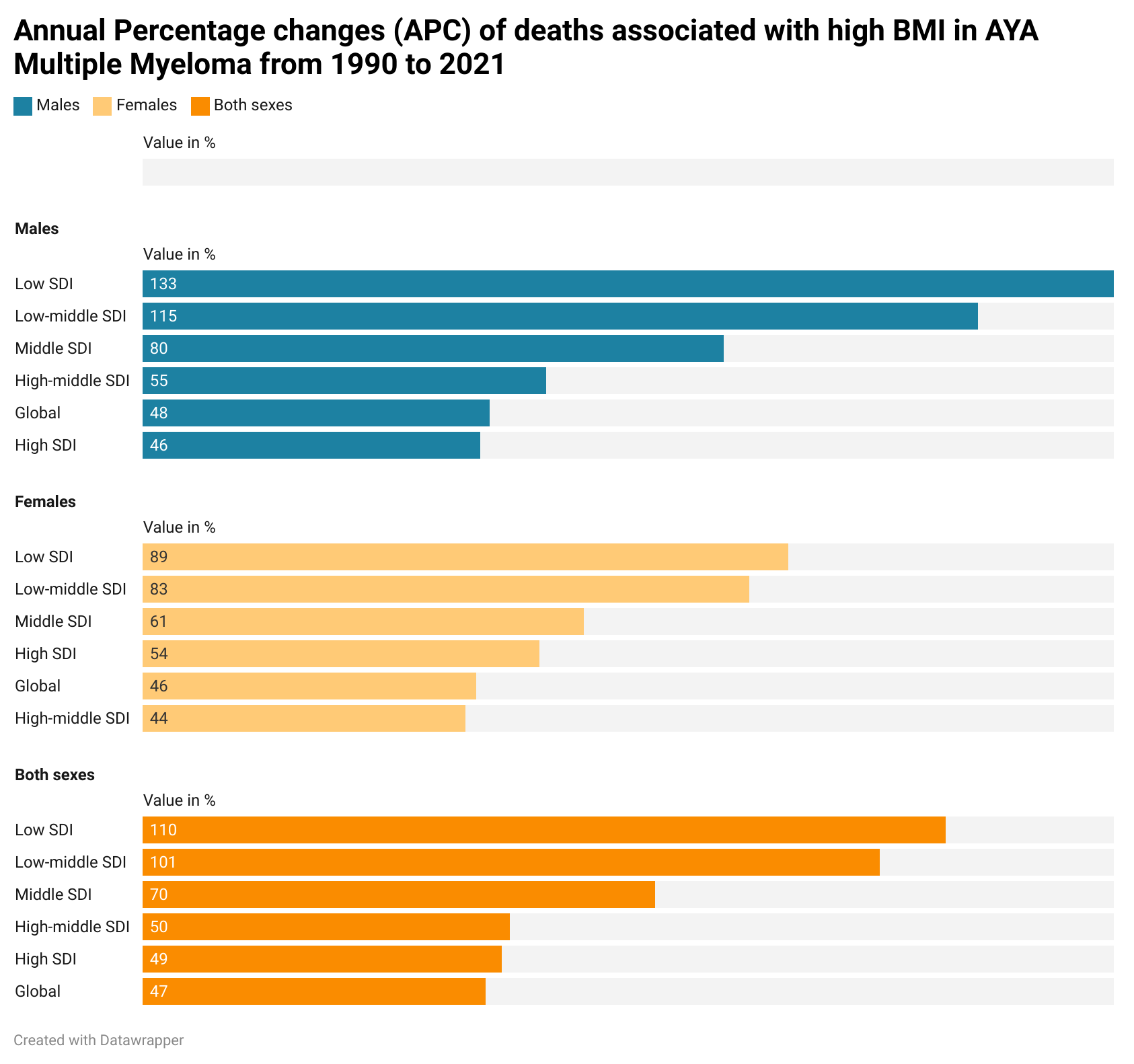


Supplemental Table 1: The Incidence Rate (IR), Death Rate (DR) in 1990 and 2021 and Annual percentage changes (APC) of incidence rate (IR) and death rate (DR) from 1990 to 2021 of adolescent and young adult (AYA) multiple myeloma in Global, Socio-demographic Index (SDI) regions, and 21 Global Burden of Disease (GBD) regions per 100,000 population among males and females.

| **Regions** | **Female 1990 IR** | **Female 2021 IR** | **Female APC IR 1990-2021** | **Male 1990 IR** | **Male 2021 IR** | **Male APC IR 1990-2021** | **Female 1990 DR** | **Female 2021 DR** | **Female APC DR 1990-2021** | **Male 1990 DR** | **Male 2021 DR** | **Male APC DR 1990-2021** |
| --- | --- | --- | --- | --- | --- | --- | --- | --- | --- | --- | --- | --- |
| Andean Latin America | 0.06 (0.05, 0.09) | 0.04 (0.02, 0.06) | 0.86 (-0.03, 2.08) | 0.10 (0.07, 0.15) | 0.07 (0.04, 0.11) | 0.59 (0.04, 1.29) | 0.05 (0.04, 0.07) | 0.03 (0.02, 0.05) | 0.45 (-0.22, 1.24) | 0.07 (0.05, 0.10) | 0.04 (0.03, 0.06) | 0.31 (-0.13, 0.85) |
| Australasia | 0.16 (0.12, 0.19) | 0.09 (0.07, 0.12) | 0.23 (-0.34, 1.31) | 0.21 (0.15, 0.30) | 0.12 (0.07, 0.21) | 0.34 (-0.09, 1.03) | 0.07 (0.06, 0.08) | 0.04 (0.03, 0.05) | -0.20 (-0.38, 0.04) | 0.06 (0.05, 0.08) | 0.03 (0.02, 0.04) | -0.13 (-0.31, 0.11) |
| Caribbean | 0.08 (0.06, 0.09) | 0.10 (0.08, 0.13) | 0.14 (-0.21, 0.58) | 0.09 (0.07, 0.11) | 0.11 (0.08, 0.15) | 0.14 (-0.13, 0.44) | 0.05 (0.05, 0.06) | 0.05 (0.05, 0.07) | 0.03 (-0.22, 0.32) | 0.06 (0.05, 0.07) | 0.06 (0.04, 0.08) | 0.05 (-0.16, 0.30) |
| Central Asia | 0.06 (0.05, 0.08) | 0.05 (0.04, 0.06) | 0.89 (0.55, 1.36) | 0.10 (0.08, 0.11) | 0.09 (0.08, 0.11) | 0.57 (0.23, 1.03) | 0.05 (0.04, 0.06) | 0.04 (0.04, 0.05) | 0.75 (0.45, 1.12) | 0.08 (0.07, 0.09) | 0.07 (0.06, 0.08) | 0.47 (0.15, 0.90) |
| Central Europe | 0.08 (0.08, 0.09) | 0.05 (0.05, 0.06) | 0.46 (0.17, 0.83) | 0.11 (0.09, 0.12) | 0.07 (0.06, 0.09) | 0.27 (0.09, 0.50) | 0.06 (0.06, 0.07) | 0.04 (0.03, 0.04) | 0.15 (-0.02, 0.32) | 0.07 (0.06, 0.07) | 0.04 (0.04, 0.05) | 0.09 (-0.04, 0.21) |
| Central Latin America | 0.07 (0.07, 0.08) | 0.05 (0.05, 0.06) | 0.68 (0.40, 1.02) | 0.12 (0.10, 0.13) | 0.09 (0.08, 0.11) | 0.64 (0.43, 0.90) | 0.06 (0.05, 0.06) | 0.04 (0.04, 0.04) | 0.37 (0.16, 0.58) | 0.08 (0.07, 0.09) | 0.06 (0.05, 0.06) | 0.39 (0.23, 0.56) |
| Central Sub-Saharan Africa | 0.02 (0.01, 0.03) | 0.01 (0.00, 0.02) | 0.31 (-0.28, 1.59) | 0.02 (0.01, 0.03) | 0.01 (0.00, 0.02) | 0.12 (-0.33, 0.65) | 0.01 (0.01, 0.02) | 0.01 (0.00, 0.01) | 0.23 (-0.32, 1.46) | 0.01 (0.01, 0.03) | 0.01 (0.00, 0.02) | 0.07 (-0.37, 0.57) |
| East Asia | 0.03 (0.02, 0.06) | 0.02 (0.02, 0.06) | 3.63 (-0.09, 8.55) | 0.20 (0.10, 0.29) | 0.11 (0.04, 0.17) | 5.47 (1.45, 10.30) | 0.03 (0.02, 0.05) | 0.02 (0.01, 0.05) | 2.31 (-0.36, 5.75) | 0.12 (0.06, 0.17) | 0.07 (0.02, 0.10) | 3.66 (0.76, 7.06) |
| Eastern Europe | 0.09 (0.08, 0.10) | 0.06 (0.06, 0.07) | 0.85 (0.50, 1.29) | 0.14 (0.13, 0.16) | 0.12 (0.10, 0.14) | 0.52 (0.29, 0.81) | 0.07 (0.06, 0.07) | 0.04 (0.04, 0.05) | 0.54 (0.28, 0.85) | 0.08 (0.08, 0.09) | 0.07 (0.06, 0.08) | 0.27 (0.09, 0.50) |
| Eastern Sub-Saharan Africa | 0.04 (0.02, 0.06) | 0.03 (0.01, 0.05) | 0.68 (-0.21, 2.56) | 0.06 (0.03, 0.10) | 0.05 (0.03, 0.08) | 0.61 (0.14, 1.55) | 0.03 (0.01, 0.05) | 0.03 (0.01, 0.04) | 0.57 (-0.26, 2.38) | 0.05 (0.03, 0.08) | 0.04 (0.02, 0.07) | 0.52 (0.07, 1.39) |
| Global | 0.05 (0.04, 0.07) | 0.04 (0.03, 0.05) | 0.79 (0.03, 1.35) | 0.10 (0.07, 0.12) | 0.07 (0.05, 0.09) | 0.88 (0.44, 1.28) | 0.04 (0.03, 0.05) | 0.03 (0.02, 0.04) | 0.58 (-0.14, 1.08) | 0.06 (0.04, 0.07) | 0.04 (0.03, 0.05) | 0.62 (0.22, 0.95) |
| High SDI | 0.11 (0.10, 0.11) | 0.07 (0.07, 0.08) | 0.28 (0.08, 0.48) | 0.14 (0.13, 0.16) | 0.10 (0.08, 0.11) | 0.34 (0.17, 0.51) | 0.07 (0.07, 0.07) | 0.04 (0.04, 0.05) | 0.03 (-0.12, 0.16) | 0.07 (0.06, 0.08) | 0.05 (0.04, 0.05) | 0.05 (-0.09, 0.16) |
| High-income Asia Pacific | 0.06 (0.05, 0.07) | 0.04 (0.03, 0.05) | 0.38 (-0.11, 1.18) | 0.07 (0.05, 0.09) | 0.05 (0.04, 0.08) | 0.12 (-0.20, 0.57) | 0.04 (0.03, 0.04) | 0.02 (0.02, 0.03) | -0.11 (-0.29, 0.07) | 0.03 (0.03, 0.04) | 0.02 (0.02, 0.02) | -0.17 (-0.32, 0.09) |
| High-income North America | 0.11 (0.11, 0.12) | 0.08 (0.07, 0.09) | -0.05 (-0.20, 0.12) | 0.10 (0.09, 0.11) | 0.08 (0.07, 0.09) | -0.10 (-0.19, 0.00) | 0.08 (0.08, 0.09) | 0.06 (0.05, 0.06) | -0.18 (-0.23, -0.13) | 0.06 (0.06, 0.07) | 0.05 (0.04, 0.05) | -0.24 (-0.28, -0.19) |
| High-middle SDI | 0.07 (0.06, 0.08) | 0.05 (0.04, 0.07) | 1.31 (0.09, 2.29) | 0.18 (0.11, 0.23) | 0.12 (0.07, 0.16) | 1.61 (0.71, 2.46) | 0.05 (0.04, 0.06) | 0.03 (0.03, 0.05) | 0.78 (-0.17, 1.52) | 0.10 (0.06, 0.12) | 0.06 (0.03, 0.08) | 1.01 (0.33, 1.61) |
| Low SDI | 0.03 (0.01, 0.04) | 0.02 (0.01, 0.04) | 0.59 (-0.12, 2.29) | 0.04 (0.02, 0.06) | 0.03 (0.02, 0.05) | 0.38 (-0.02, 1.26) | 0.02 (0.01, 0.04) | 0.02 (0.01, 0.03) | 0.49 (-0.17, 2.08) | 0.03 (0.02, 0.05) | 0.03 (0.02, 0.04) | 0.30 (-0.07, 1.14) |
| Low-middle SDI | 0.03 (0.02, 0.05) | 0.02 (0.01, 0.04) | 1.12 (0.08, 2.50) | 0.06 (0.04, 0.08) | 0.05 (0.03, 0.07) | 0.88 (0.39, 1.92) | 0.03 (0.01, 0.04) | 0.02 (0.01, 0.03) | 0.93 (-0.03, 2.19) | 0.04 (0.03, 0.06) | 0.04 (0.03, 0.05) | 0.72 (0.27, 1.67) |
| Middle SDI | 0.04 (0.03, 0.05) | 0.03 (0.02, 0.05) | 1.51 (0.10, 2.38) | 0.11 (0.07, 0.14) | 0.07 (0.05, 0.10) | 1.89 (0.87, 2.77) | 0.03 (0.02, 0.04) | 0.02 (0.02, 0.04) | 1.06 (-0.12, 1.82) | 0.07 (0.05, 0.09) | 0.05 (0.03, 0.06) | 1.37 (0.53, 2.10) |
| North Africa and Middle East | 0.05 (0.03, 0.07) | 0.04 (0.02, 0.09) | 0.85 (-0.15, 2.34) | 0.09 (0.06, 0.12) | 0.08 (0.05, 0.11) | 0.71 (0.23, 1.40) | 0.04 (0.02, 0.06) | 0.03 (0.02, 0.07) | 0.44 (-0.30, 1.52) | 0.06 (0.04, 0.07) | 0.05 (0.03, 0.07) | 0.35 (-0.00, 0.88) |
| Oceania | 0.02 (0.01, 0.04) | 0.03 (0.02, 0.06) | 0.60 (0.02, 1.74) | 0.02 (0.01, 0.04) | 0.05 (0.02, 0.08) | -0.07 (-0.34, 0.32) | 0.02 (0.01, 0.03) | 0.02 (0.01, 0.05) | 0.56 (-0.02, 1.71) | 0.02 (0.01, 0.03) | 0.04 (0.02, 0.07) | -0.08 (-0.33, 0.32) |
| South Asia | 0.04 (0.02, 0.06) | 0.03 (0.01, 0.04) | 1.07 (-0.02, 3.05) | 0.07 (0.05, 0.10) | 0.05 (0.03, 0.08) | 0.78 (0.21, 2.20) | 0.03 (0.02, 0.05) | 0.02 (0.01, 0.03) | 0.85 (-0.12, 2.64) | 0.05 (0.04, 0.08) | 0.04 (0.02, 0.06) | 0.60 (0.08, 1.86) |
| Southeast Asia | 0.02 (0.01, 0.03) | 0.01 (0.01, 0.02) | 0.90 (0.02, 1.89) | 0.03 (0.02, 0.06) | 0.03 (0.02, 0.04) | 0.90 (0.36, 1.71) | 0.01 (0.01, 0.02) | 0.01 (0.01, 0.02) | 0.68 (-0.10, 1.56) | 0.02 (0.02, 0.04) | 0.02 (0.01, 0.03) | 0.69 (0.22, 1.43) |
| Southern Latin America | 0.10 (0.08, 0.11) | 0.06 (0.05, 0.08) | 0.06 (-0.25, 0.46) | 0.13 (0.11, 0.16) | 0.07 (0.05, 0.09) | 0.36 (0.07, 0.80) | 0.07 (0.06, 0.08) | 0.05 (0.04, 0.06) | -0.17 (-0.35, 0.07) | 0.07 (0.07, 0.09) | 0.04 (0.03, 0.05) | 0.07 (-0.12, 0.32) |
| Southern Sub-Saharan Africa | 0.11 (0.07, 0.15) | 0.09 (0.05, 0.12) | 0.53 (-0.16, 1.98) | 0.18 (0.11, 0.25) | 0.14 (0.08, 0.23) | 0.56 (0.10, 1.21) | 0.09 (0.06, 0.12) | 0.07 (0.04, 0.10) | 0.45 (-0.19, 1.77) | 0.13 (0.08, 0.19) | 0.10 (0.06, 0.17) | 0.47 (0.06, 1.07) |
| Tropical Latin America | 0.07 (0.06, 0.07) | 0.05 (0.05, 0.06) | 1.02 (0.74, 1.34) | 0.13 (0.12, 0.14) | 0.11 (0.10, 0.12) | 0.93 (0.72, 1.15) | 0.05 (0.05, 0.06) | 0.04 (0.04, 0.04) | 0.69 (0.51, 0.85) | 0.09 (0.08, 0.09) | 0.07 (0.06, 0.07) | 0.68 (0.52, 0.83) |
| Western Europe | 0.14 (0.13, 0.16) | 0.10 (0.09, 0.12) | 0.11 (-0.14, 0.42) | 0.19 (0.16, 0.22) | 0.11 (0.09, 0.14) | 0.31 (0.12, 0.54) | 0.07 (0.07, 0.08) | 0.05 (0.04, 0.05) | -0.22 (-0.29, -0.16) | 0.06 (0.06, 0.07) | 0.04 (0.03, 0.04) | -0.13 (-0.20, -0.05) |
| Western Sub-Saharan Africa | 0.01 (0.01, 0.02) | 0.01 (0.00, 0.01) | 1.07 (0.00, 2.62) | 0.02 (0.01, 0.02) | 0.02 (0.00, 0.03) | 0.59 (0.04, 1.49) | 0.01 (0.00, 0.01) | 0.01 (0.00, 0.01) | 0.93 (-0.05, 2.31) | 0.01 (0.01, 0.02) | 0.01 (0.00, 0.02) | 0.49 (-0.02, 1.27) |
